# Novel Progress in Bat Biological Research: Evolution, Physiology, Behavior and Conservation

**DOI:** 10.3390/biology15141096

**Published:** 2026-07-08

**Authors:** Jiang Feng, Ying Liu, Hui Wang

**Affiliations:** 1 Jilin Provincial Key Laboratory of Animal Resource and Ecological Security, Northeast Normal University, Changchun 130117, China; 2 College of Life Science, Jilin Agricultural University/Cultivation Base of State Key Laboratory for Ecological Restoration and Ecosystem Management of Jilin Province, Changchun 130118, China; 3 Jilin Provincial International Cooperation Key Laboratory for Biological Control of Agricultural Pests, Jilin Agricultural University, Changchun 130118, China

Bats (order Chiroptera) are among the most diverse and ecologically vital mammals, defined by powered flight, remarkable echolocation, and exceptional physiological adaptations. Recent interdisciplinary progress has deepened our understanding of bat evolution, physiology, and behavior, yet several key limitations remain, particularly with regard to sensory mechanisms, developmental dynamics, and conservation responses [1,2,3,4,5,6,7,8,9].

This Special Issue unites 13 original studies that address these challenges, spanning molecular evolution [10,11,12,13], developmental morphology [14,15], bioacoustics [12,16], behavioral ecology [17,18], and conservation science [19,20,21,22]. Collectively, the work advances our knowledge of bat biology across scales, from molecular pathways to ecosystem interactions.

In evolutionary research, comparative transcriptomics of closely related *Rhinolophus* species reveals that echolocation divergence arises primarily from transcriptional regulation of muscle and metabolic genes, rather than coding mutations [12]. Phylogenomic analyses further resolve cryptic diversity in the *Rhinolophus macrotis* group, clarifying unresolved questions about recent speciation [11].

Developmental studies, using 3D scanning and geometric morphometrics, document postnatal skull dynamics in *Vespertilio sinensis*, linking morphological plasticity to feeding adaptation [14]. Bioacoustic work combines machine learning and playback experiments, demonstrating that *Rhinolophus* distress calls encode sex, age, and individual signatures, enabling social recognition [16].

Physiological research on bat forehead gland chemosignals demonstrates that colony odor divergence is uncoupled from symbiotic microbiota [13], while field studies highlight bats’ critical role in agricultural pest control [21,22]. Conservation contributions provide habitat models and molecular tools, improving non-invasive monitoring and baseline assessments for threatened populations [19].

In summary, this Special Issue synthesizes interdisciplinary progress, remedying prior limitations and offering actionable insights. While advances are substantial, broader genomic sampling, long-term ecological monitoring, and climate impact assessments remain priorities. We hope this collection stimulates continued collaboration, supporting both fundamental research and conservation of these remarkable mammals.

We thank all authors, reviewers, and the Editorial Office for their support.

## References

[1] Amichai E., Yovel Y. (2021). Echolocating bats rely on an innate speed-of-sound reference. Proc. Natl. Acad. Sci. USA.

[2] Chen S., Yovel Y., Moss C.F., Luo J. (2026). Evolution of vocal control in echolocating bats. Biol. Rev..

[3] Gardner N.M., Dececchi T.A. (2022). Flight and echolocation evolved once in Chiroptera: Comments on ‘The evolution of flight in bats: A novel hypothesis’. Mammal Rev..

[4] Jebb D., Huang Z., Pippel M., Hughes G.M., Lavrichenko K., Devanna P., Winkler S., Jermiin L.S., Skirmuntt E.C., Katzourakis A. (2020). Six reference-quality genomes reveal evolution of bat adaptations. Nature.

[5] Li A., Zhang H., Lu M., Cao Q., Tian X., Luo J. (2026). Robust Doppler Shift Compensation in Freely Flying Bats Under Acoustic Interference. Integr. Zool..

[6] Nojiri T., Wilson L.A.B., López-Aguirre C., Tu V.T., Kuratani S., Ito K., Higashiyama H., Son N.T., Fukui D., Sadier A. (2021). Embryonic evidence uncovers convergent origins of laryngeal echolocation in bats. Curr. Biol..

[7] Norberg U.M., Rayner J.M. (1987). Ecological morphology and flight in bats (Mammalia; Chiroptera): Wing adaptations, flight performance, foraging strategy and echolocation. Philos. Trans. R. Soc. B Biol. Sci..

[8] Usui K., Yamamoto T., Khannoon E.R., Tokita M. (2024). Musculoskeletal morphogenesis supports the convergent evolution of bat laryngeal echolocation. Proc. R. Soc. B Biol. Sci..

[9] Yoshida S., Mastumoto H., Kobayasi K.I., Hiryu S. (2026). Horseshoe bats (*Rhinolophus nippon*) suppress clutter noise through echolocation frequency control to detect prey. Commun. Biol..

[10] Zhu Y., Liu S., Du J., Xiao Y., Sun K. (2026). Hepatic Transcriptome Variations Among Different Evolutionary Lineages of *Rhinolophus ferrumequinum* During Hibernation. Biology.

[11] Cong J., Zhang J., Yu H., Lei J., Miao G., Yang H., Li Q., Zhang Z., Csorba G., Sun K. (2026). Integrative Taxonomy Reveals a Candidate Lineage Within the *Rhinolophus macrotis* Group. Biology.

[12] Miao G., Cong J., Lei J., Quan S., Li J., Li Y., Zhang K., Liu T. (2026). Laryngeal Transcriptomic Insights into Echolocation Call Frequency Divergence in Closely Related *Rhinolophus* Species. Biology.

[13] Zheng Z., Lucas J.R., Zhang C., Sun C. (2026). Do Symbiotic Microbes Drive Chemical Divergence Between Colonies in the Pratt’s Leaf-Nosed Bat, *Hipposideros pratti*?. Biology.

[14] Li X., Bao M., Chang Y., Wang H., Feng J. (2025). Novel 3D Scanning and Multi-Angle Analysis Uncover the Ontogenetic Developmental Dynamics of the Skull in *Vespertilio sinensis*. Biology.

[15] Zhang M., Wang H., Liu Z., Bao M., Li X., Wang T., Wang R., Feng J. (2025). What Factors Shape the Flyability in Bats?—The Perspective from Bat’s Wing Development. Biology.

[16] Hao J., Li J., Wang B., Guo M., Tan X., Zhang K. (2026). Acoustic Features and Recognition of Distress Calls in Rhinolophus nippon: A Study Combining Machine Learning and Playback Experiments. Biology.

[17] Vivanco-Montané O.R., Morales-Mávil J.E., Hernández-Salazar L.T., Pérez-Torres J., Bello-Sánchez E.A. (2025). Warning Before a Fight: The Role of Distance and Ritualized Agonistic Behaviors in Minimizing Aggression in the Jamaican Fruit Bat. Biology.

[18] Pergams A., Haus M., Estrada C., Brown J., Salles A. (2025). Dinner Date: Opposite-Sex Pairs of Fruit Bats (*Rousettus aegyptiacus*) Forage More than Same-Sex Pairs. Biology.

[19] Liu Y., Geng Y., Pan Y., Zeng H., Huang Z., Taylor P.J., Jiang T. (2025). Climate-Driven Shifts in Bat Distributions Reveal Functional Reorganization and Spatial Mismatch Across Agroecosystems. Biology.

[20] Wang F., Song W., Wang D., Huang Z., Shan M., Sun S., Jin Z., Lu J., Ji Y., Sun K. (2025). Investigating the Dynamic Variation of Skin Microbiota and Metabolites in Bats During Hibernation. Biology.

[21] Guo Q., Liu Y., Liu S., Geng Y. (2026). Research Progress in Bat Dietary Analysis: Methods, Applications, and Future Perspectives. Biology.

[22] Abdullah N.-I., Elias N.-A., Ohte N., Vincenot C.E. (2026). Bat Community Response to Insect Abundance in Relation to Rice Phenology in Peninsular Malaysia. Biology.

